# Relationship of gene markers to residual feed intake, ADG, and marbling of 4 years of MSU Steer-A-Year steers^1^

**DOI:** 10.1093/tas/txaa131

**Published:** 2020-12-22

**Authors:** Makae F Nack, Hannah M DelCurto Wyffels, Samuel A Wyffels, Timothy DelCurto

**Affiliations:** 1 Northern Agricultural Research Center, Montana State University, Havre, MT; 2 Department of Animal and Range Sciences, Montana State University, Bozeman, MT

## INTRODUCTION

In beef cattle production, producers are constantly looking for ways to improve their bottom line. Feed costs can make up to 80% of operation costs for beef producers (Kelly et al., 2011). Improving average daily gain (ADG) and residual feed intake (RFI) can help reduce feed costs. Increasing profits by knowing how animals will finish based on carcass traits is another tool that can be used by operations to improve the efficiency of production. Producing cattle that will return the most profit is essential for a successful operation. Advancements in technology and use of information from the beef genome have provided additional tools for producers wanting to enhance the production of their cattle. The Merial Ltd (Duluth, GA) Igenity provides a genetic test that produces information to predict calf performance. Selection for cattle that are more efficient or attain ideal grades can be based off these predictions.

Previous studies have determined a high correlation between genotypic and phenotypic feed efficiency in growing Charolais bulls (Arthur et al., 2001). Predicted and actual ADG have been correlated in a feedlot setting (Thompson et al., 2014). Van Eenennaam et al. (2007) determined a significant association between quality grade and the TG5 allele in Charolais × Angus crosses. High accuracy of genetic testing can greatly benefit those who use it, allowing operations to select for cattle who are more efficient. Genetic testing is commonly used in seedstock industries but is not as readily used in commercial or feedlot cattle. Accurately using genetic markers to predict phenotypic characteristics across breeds and environments can be difficult (Van Eenennaam et al., 2007). This may be due to breed variation, environmental differences, or inherent inaccuracy with the genetic markers. Thus, the objective of this study was to evaluate the accuracy and correlation of genotypic and phenotypic feed efficiency and carcass quality in feedlot cattle. With this information, we hope to further our understanding of the Igenity tests application to a commercial feedlot setting.

## MATERIAL AND METHODS

Experimental procedures described herein were approved by the Agriculture Animal Care and Use Committee of Montana State University (MSU; #2016-AA01). Calves were finished at Bozeman Agriculture Research and Teaching Farm (BART Farm) in Bozeman, MT. In the Steer-A-Year program, 118 weaned steer calves ranging from 9 to 11 months of age upon arrival were fed over a 4-yr period (2015–2016, *n* = 25; 2016–2017, *n* = 32; 2017–2018, *n* = 28; 2018–2019, *n* = 33) for an average of 6 months (December–June). Calves were donated from producers across Montana and were comprised of various breeds; however, the majority were Angus based. Upon arrival, calves were tagged with an RFID (Allflex USA Inc., Dallas/Ft. Worth Airport, TX). In addition, calves were implanted with Component T-S (Elanco Animal Health, Greenfield, IN) at the beginning of the feeding period. Steers were penned in three drylot pens, fenced with wooden-slatted fencing. Calves in each drylot had access to water in the center of the pens and a shelter at the north end. Each pen was equipped with two GrowSafe Intake units (GrowSafe Systems Ltd., Airdrie, AB, Canada) for measuring individual animal intake. Calves were assigned to pens based on initial weight (light, middle, and heavy weights). The number of calves per pen varied between 8 and 12 head (4–6 head per GrowSafe unit). Steers were started on a ration of 50% coarsely chopped hay, 5% 40-20 Beef Finisher supplement (CHS Inc., Sioux Falls SD), and 45% cracked corn. Through January, corn increased in 5% increments once a week and, by mid-February, the calves were on a full ration of 20% coarsely chopped hay, 5% 40-20 Beef Finisher supplement, and 75% cracked corn. Steers had ad libitum access to feed and were fed twice daily at 0800 and 1700 hours. Sweetlix Bloat Guard (Ridley USA Inc., Mankato MN) was available to steers on an ad libitum basis.

Steers were weighed at the start of feeding for an initial weight and every 28 d until slaughter. Ear tissue samples from each calf were sent to Igenity (Merial Ltd Duluth, GA) for analysis. For many of the performance traits predictions, Igenity placed the values into a relative index (1–10). For most traits, higher scores are more desirable. Carcass measurements were attained at a local abattoir after slaughter.

RFI was calculated for years 2–4 using the method developed by Arthur et al. (2001). Igenity score and observed performance and carcass traits were evaluated using Pearson product–moment correlation test. In addition, Spearman rank correlation was also used to determine if the phenotypic and genotypic measurements ranked the animals in a similar order. An α ≤ 0.05 was considered a significant relationship. All data were analyzed in R (R Core Team 2017).

## RESULTS

**Figure 1.**
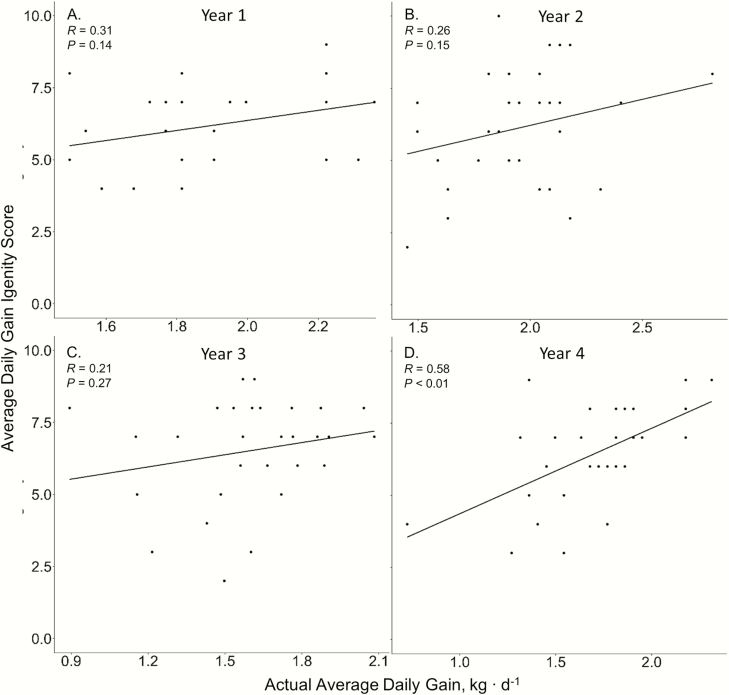
Relationship of steer ADG and Igenity ADG score across 4 years.

**Figure 2.**
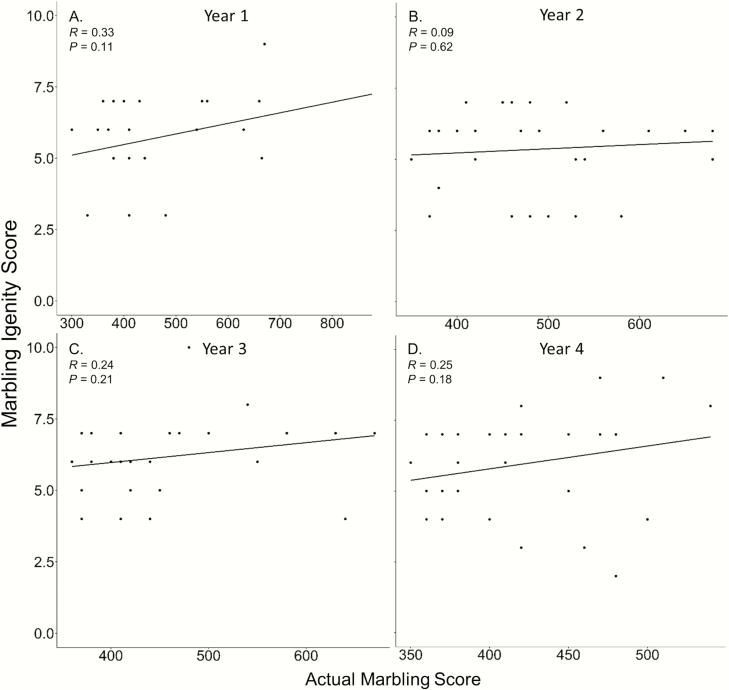
Relationship of steer marbling score and Igenity marbling score across 4 years.

**Figure 3.**
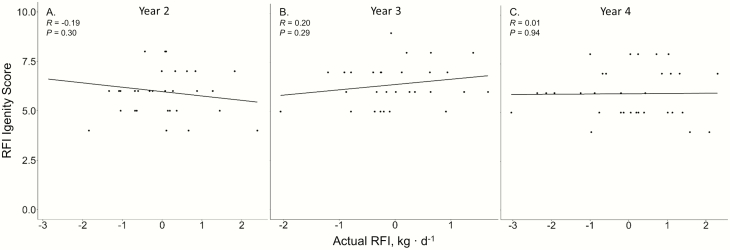
Relationship of steer calculated RFI and Igenity RFI score across 4 years.

Igenity scores were not related to actual steer ADG for years 1, 2, and 3 (*P* ≥ 0.14; Fig. 1A–C). In addition, phenotypic ADGs were not ranked in a similar order as the ADG Igenity scores in years 1, 2, and 3 (*P* ≥ 0.14; Table 1). However, Igenity scores were positively correlated to steer ADG (*P* < 0.01; *r* = −0.58; Fig. 1D) and ranked similarly in year 4 (*P* < 0.01; ρ = 0.55; Table 1). Steer RFI displayed no relationship to the Igenity RFI score (*P* ≥ 0.29; Fig. 2A–C). Furthermore, the Igenity RFI score did not rank steers in a similar order to calculated RFI (*P* ≥ 0.31; Table 1). No linear relationships were observed between the Igenity marbling score and actual marbling in any year (*P* ≥ 0.11; Fig. 3A–D). Additionally, Igenity scores did not rank steers similarly to actual marbling scores in year 1 and 2 (*P* ≥ 0.21; Table 1) but did display tendencies to rank animals similar to actual marbling in years 3 and 4 (*P* ≤ 0.08; ρ = 0.34, 0.31, respectively; Table 1).

**Table 1.** Spearman correlation coefficients (ρ) for paired associations between Igenity score and phenotypic traits of feedlot steers over 4 years

	ρ	*P*-value
ADG		
Year 1	0.31	0.14
Year 2	0.2	0.26
Year 3	0.2	0.31
Year 4	0.55	<0.01
Marbling		
Year 1	0.27	0.21
Year 2	0.07	0.7
Year 3	0.34	0.07
Year 4	0.31	0.08
RFI		
Year 2	−0.07	0.74
Year 3	0.19	0.31
Year 4	−0.04	0.82

## DISCUSSION

Similar to our study, weak correlation and no statistical significance for predicted versus actual estimates for RFI have been reported by other researchers (Damiran et al., 2018). However, in our study, year 4 of ADG had a correlation between predicted and actual. This may suggest improvements in technology and more robust databases. Similar to our study, other researchers have reported a correlation between actual and predicted ADG; however, overall correlation between Igenity panels and actual performance was low (DeVuyst et al., 2011). Regardless, the accuracy of the genetic prediction of production traits still requires further research and validation. Additionally, genetics is complex, one gene can influence multiple traits and phenotypic characteristics (Van Eenennaam et al., 2007). Furthermore, our study suggests that using Igenity to accurately predict marbling, RFI, and, to an extent, ADG, for mix-breed feedlot cattle is limited. Further research needs to be done to validate Igenity and using genetic markers for selection across breeds and environments in a feedlot setting.
